# The Role of Proteomics in Identification of Key Proteins of Bacterial Cells with Focus on Probiotic Bacteria

**DOI:** 10.3390/ijms25168564

**Published:** 2024-08-06

**Authors:** Miroslava Stastna

**Affiliations:** Institute of Analytical Chemistry of the Czech Academy of Sciences, Veveri 97, 602 00 Brno, Czech Republic; stastna@iach.cz

**Keywords:** bacterial proteins, probiotics, mass spectrometry-based proteomics, health-promoting effect

## Abstract

Probiotics can affect human health, keep the balance between beneficial and pathogenic bacteria, and their colonizing abilities enable the enhancement of the epithelial barrier, preventing the invasion of pathogens. Health benefits of probiotics were related to allergy, depression, eczema, cancer, obesity, inflammatory diseases, viral infections, and immune regulation. Probiotic bacterial cells contain various proteins that function as effector molecules, and explaining their roles in probiotic actions is a key to developing efficient and targeted treatments for various disorders. Systematic proteomic studies of probiotic proteins (probioproteomics) can provide information about the type of proteins involved, their expression levels, and the pathological changes. Advanced proteomic methods with mass spectrometry instrumentation and bioinformatics can point out potential candidates of next-generation probiotics that are regulated under pharmaceutical frameworks. In addition, the application of proteomics with other omics methods creates a powerful tool that can expand our understanding about diverse probiotic functionality. In this review, proteomic strategies for identification/quantitation of the proteins in probiotic bacteria were overviewed. The types of probiotic proteins investigated by proteomics were described, such as intracellular proteins, surface proteins, secreted proteins, and the proteins of extracellular vesicles. Examples of pathological conditions in which probiotic bacteria played crucial roles were discussed.

## 1. Introduction

The term “probiotics” was revisited in 2014, and probiotics were defined as “live microorganisms that, when administered in adequate amounts, confer a health benefit on the host” [1,2]. Generally, probiotics can be considered as good or beneficial bacteria and can include Gram-positive (single cell membrane—monoderm prokaryotes) or Gram-negative (two distinct membranes—diderm prokaryotes) types [3] that differ in both the structure of the cell surface and the surface proteins, and thus, in their interactions with the environment [4,5]. The most often probiotic bacteria are *Lactobacillus* and *Bifidobacterium* [6,7,8] with other types known, such as *Bacillus*, *Streptococcus*, *Propionibacterium*, *Saccharomyces* [9], and *Pediococcus* [10]. The strains of *Lacticaseibacillus rhamnosus* (*L. rhamnosus* GG) and *Lactiplantibacillus plantarum* (*L. plantarum* WCFS1) belong to the most commercially used and clinically examined probiotic microorganisms with potential for the treatment of a variety of disease conditions if given to patients, e.g., as supplements [6,11,12,13].

The efficiency of probiotics depends on the interaction of probiotic bacteria with gut microbiota (a population of microorganisms that lives/colonizes in the digestive tract) of the host and in the enhancement of the growth of beneficial microorganisms and the inhibition of pathogenic microorganisms [14,15]. The bacterial imbalance in the gut (dysbiosis) is connected to various pathologies. Moreover, the effect of probiotics on microbiota is species/strains specific and thus, probiotics regulate physiological functions in different ways [15]. For example, the interplay between gut microbiota and brain function (gut-brain axis) was highlighted recently, and novel multi-strain E3 probiotic formulation was shown to remodel gut microbiota and improve mental health symptoms in the population with depression, anxiety, and sleep problems [16].

**Figure 1:**
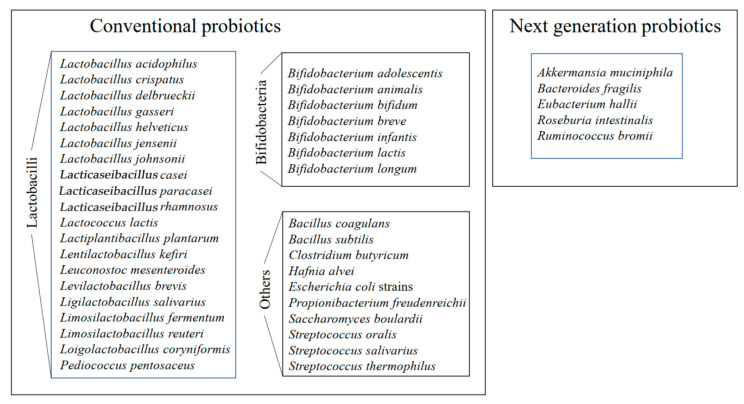
The list of conventional probiotic bacteria and next-generation probiotics that have the potential for health prevention and disease treatment. This figure was adapted from ref. [9].

Probiotic bacteria have been shown to affect human health in a positive way via diverse mechanisms, for example, by enhancing the uptake of dietary nutrients, improving digestion, altering the gastric pH, improving the epithelial barrier function [17,18], protecting against physiological stress, strengthening the protection and reaction against pathogens, and regulating the immune response [19,20,21,22,23]. For example, lactic acid bacteria, with their probiotic properties, can maintain gut homeostasis by regulating inflammatory and immune reactions. Many probiotics can function as microbial barriers against pathogenic bacteria via competitive binding and/or production of inhibitory compounds that decrease the proliferation of pathogens [24]. For example, the production of protective proteinaceous compounds bacteriocins in vivo in a mice model was registered during anti-infective activity of *Ligilactobacillus salivarius* against pathogen *Listeria monocytogenes* [25], and the production of lactic acid was demonstrated during antibacterial and inhibition functions of six lactobacilli against invasion of *Salmonella enterica* into human enterocyte-like cells [26]. Moreover, promising potential candidates of next-generation probiotics such as *Akkermansia muciniphila*, *Bacteroides fragilis*, *Roseburia intestinalis*, and *Eubacterium hallii* have been studied for their beneficial properties for human health [1,27,28,29,30,31]. The list of probiotic bacteria and next-generation probiotics that have the potential for health prevention and disease treatment was compiled in Figure 1.

The recognition of molecular mechanisms of probiotic activities, the knowledge about how health-promoting probiotic bacteria induce their effects, and the identification of probiotic molecules of action (effector molecules) are the challenges that need to be examined in detail [32]. The complexity of live microorganisms that can contain a great number of bioactive molecules should be characterized, and multiple interactions between probiotics and their hosts should be explained [22]. Examples of effector molecules are surface-located molecules (long pili structures, S-layer proteins, exopolysaccharides, muropeptides) and various metabolites and enzymes (p. 40 and p. 75) [33,34,35]. It was demonstrated that soluble proteins produced by probiotic bacteria have protective and preventive effects on intestinal epithelial cells during oxidant stress [36], survival and growth [35], and intestinal inflammatory diseases [37].

Proteomics, mass spectrometry (MS), and bioinformatic developments in recent years entail a great potential for novel discoveries in the field of probiotics, for characterization of the molecular mechanisms underlying probiotic effects, and for recognition of dynamics during interaction between the proteins of probiotic bacteria and the immune/epithelial cells of the intestinal tract [7,18]. It can provide information about the types of proteins involved, their expression levels, and their pathological changes. The number of proteomic studies reported in the probiotics field is still insufficient despite recent advancements in proteomic methodologies and instrumentation. Nevertheless, proteomic approaches have been applied over the years to better understand the beneficial effects of probiotic bacteria. Review articles have been published highlighting the usefulness of proteomics, for example, for investigation of the bacterial ability to adapt to the harsh environment in the gastro-intestinal tract (bile tolerance, low pH, etc.) and the adhesion to host epithelial cells [7], for analysis of probiotic strains [38], for evaluation of probiotic functionality [39], and within the genus *Lactobacillus* [40].

In this review, the proteomic strategies for identification/quantitation of the proteins that comprise the probiotic bacterial cells were overviewed and the types of probiotic proteins investigated by proteomics were described, such as intracellular proteins, surface proteins, extracellular (secreted) proteins, and the proteins of extracellular vesicles derived from probiotic bacterial cells. Finally, examples of pathological conditions in which probiotic bacteria played crucial roles were discussed. Focus was placed on the studies that were reported most recently; however, earlier discoveries were mentioned as well. Although the most often applications of probiotics were directed to gastrointestinal disorders, this review targeted the roles of probiotics in a variety of other pathologies. The modulation of gut intestinal microbiota and their examination through proteomics could be found in published reviews [15,38,39,41].

## 2. Proteomic Methods, Including MS-Based Approaches

**Figure 2:**
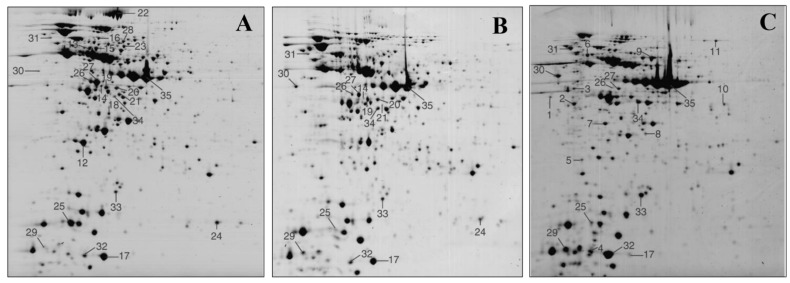
Representative 2-DE gel patterns and the differences in the expression of the proteins produced by three *Lactobacillus plantarum* strains. (**A**) *L. plantarum* 299v; (**B**) *L. plantarum* CECT 4185; (**C**) *L. plantarum* WHE 92. The pH range was 4–7, the gels were stained by Coomassie blue, and 75 μg of total protein were loaded. In total, 35 protein spots (marked in the gels) that exhibited differential intensities between compared gels were excised from the gels, and the proteins in the spots were identified by mass spectrometry. The figure was adapted from ref. [45] with permission.

The classical proteomic methods for analysis of probiotic bacterial cells included fractionation of the proteins by one- or two-dimensional gel electrophoresis (1-DE or 2-DE) followed by protein identification/quantitation by MS techniques (Figure 2), such as matrix-assisted laser desorption/ionization-time of flight (MALDI TOF/TOF MS) or nano liquid chromatography coupled to tandem mass spectrometer via electrospray ionization (LC-MS/MS) [42,43,44,45] with subsequent search and match of MS spectra against protein databases. However, there are limitations using 2-DE, such as difficulty to solubilize hydrophobic proteins and poor detectability of low-abundance proteins. Nevertheless, the bacterial proteins can be analyzed directly by using LC-MS/MS in bottom-up (shotgun) proteomic approach, i.e., after their digestion by various enzymes or their combination and without gel pre-separation. Usually, data-dependent acquisition (DDA) is applied in LC-MS/MS, which means that first, the survey scan is performed on the sample with digested peptides in the LC-MS/MS instrument, followed by selection of the most intense peptide precursor ions for subsequent MS/MS fragmentation. For LC-MS/MS protein quantification, strategies such as label-free methods and metabolic labeling techniques can be used. In label-free quantification, two approaches can be used, i.e., spectral counting method, in which the number of MS/MS spectra assigned to a particular protein are counted across different samples, and spectral ion currents (signal intensities) method, in which the intensities of chromatographic peaks of the same peptide between different samples are measured. In addition, specific methodologies have been developed for analysis of probiotic bacterial surface-exposed proteins and the proteins secreted by bacteria [46].

The crucial step to fully utilize the potential of highly sensitive MS instrumentation is the preparation of quality samples for analysis. Recently, the workflow efficiency was evaluated in terms of sample preparation, MS data acquisition, and MS data analysis for six bacterial species which represented diversity in various features [47]. Among the experimental conditions tested, a unified proteomic workflow was found that incorporated the lysis of bacterial cells with a 100% trifluoroacetic acid (TFA), in solution digestion of the proteins and separation of digested peptides by 30-min microflow liquid chromatography followed by MS performed in data independent acquisition mode. The workflow feasibility was confirmed for 23 different bacterial species with over 45,000 proteins identified in the MS combined dataset; out of them, 30,000 proteins were not detected previously [47].

The analysis of membrane proteins, including surface proteins of probiotic bacterial cells, is generally challenging since the proteins are present in low abundances, they are difficult to dissolve and prepare without contamination from other non-surface proteins. In addition, the use of detergents to solubilize the membrane proteins should be avoided since they interfere with downstream MS identification. Thus, cell surface shaving and cell surface labeling techniques were applied [19,48,49] to obtain enriched preparations of bacterial surface proteins. Although often applied for various pathological bacterial cells, these strategies are applicable for surface proteins of probiotic bacteria as well. By use of cell surface shaving, the exposed regions of surface-exposed proteins were enzymatically cleaved (shaved) by incubation of intact cells with, e.g., trypsin under short time periods and isotonic conditions to ensure that the cell rupture was minimized. On contrary to the hydrophobicity of membrane proteins, their exposed parts were usually hydrophilic, which allowed their digestion in aqueous buffers [48]. This resulted in the mixture of peptides that originated from surface-exposed proteins which were further analyzed and identified by LC-MS/MS. To minimize false-positive identifications by cytosolic proteins contamination that could result from compromised cell stability during enzymatic treatment [50], the subtracted strategy was reported that included a false-positive control set. This process was shown to improve the accuracy of cell surface peptides in the given dataset up to 80% (124 from 155 peptides) [50]. Using cell surface labeling techniques, surface-exposed proteins of intact cells were labeled by, e.g., biotinylation reagent that was able to label even the proteins within the cell wall. Following labeling, the cells were subjected to lysis, the labeled surface proteins were enriched by affinity chromatography using biotin-streptavidin chemistry, and eluted surface proteins were identified by MS-based proteomic workflow [19,49].

The proteins secreted by bacterial cells (bacterial secretomes) were usually identified by analysis of media in which the cells were conditioned in vitro using MS-based protein identification/quantitation. Thus, various culture conditions and/or mutant strains were studied to recognize and characterize the proteins that were involved in particular secretion machinery [46]. Since the secretion of the proteins by the cells as a response to various physiological and environment stimuli is highly dynamic, the metabolic labeling methods used in dynamic proteomics, such as SILAC [51] and non-canonical labeling [52,53], have been developed for secreted protein identification and quantitation both in vitro and in vivo. These can be promising techniques for application to probiotic bacterial cells in the near future. In the non-canonical labeling approach, methionine surrogates AHA (azidohomoalanine) or HPG (homopropargylglycine) were used as labels that incorporated into proteins at the time of their synthesis both in vitro and in vivo, and thus, this method allowed the detection of the changes in protein abundances soon after their synthesis. This is the advantage compared to the SILAC method, in which several cell divisions are needed for full incorporation of the label. As well, the enrichment step that is included in non-canonical labeling techniques enabled the identification of synthesized proteins with low abundances. The labeled proteins that were newly synthesized due to various pathological conditions could be distinguished from the pre-existing protein pool, and the protein candidates can be pointed out for targeted treatment and early recognition of protein dysregulation.

The proteins obtained from MS identification need to be further validated using various targeted assays such as Western blotting and enzyme-linked immunosorbent assay (ELISA). However, a sensitive and accurate MS-based antibody-free assay called multiple/selected reaction monitoring (MRM/SRM) can be used for protein validation and quantification when antibody is not available [54]. As well, proteomic data can be validated by other biochemical/physiological methods to gather additional information and obtain a more complete view of the studied systems.

## 3. Proteins of Probiotic Bacterial Cells and the Examples Studied by Proteomics

Probiotic bacterial cells contain many proteins, either the proteins embedded or anchored to the cell surface or intracellular proteins, which can be secreted into the extracellular environment. In addition, probiotic cells produce extracellular vesicles (EVs) containing various proteins, and these EVs were shown to be the potential structures for pro-health implementation.

### 3.1. Whole Cell Lysates and Intracellular Proteins Identification

In the past, comparative proteomic analysis was applied to nine *Lactobacillus plantarum* strains in vitro that exhibited different bile tolerance properties and levels (sensitive, intermediate, and resistant) [55]. Herein, the strains were subjected to bile stress using increased Oxgall concentrations (0.5–3.6%). The characteristic proteome profiles were identified for individual strains, and key proteins were pinpointed that could be used as bacterial biomarkers for future selection of the strain with optimal probiotic functions [55].

Recently, four strains of *Lactococcus lactis* (subsp. *lactis* NCDO2118, *lactis* IL1403, *cremoris* NZ9000, and *cremoris* MG1363) were characterized by a comparative proteomic approach [56]. The label-free quantitative analysis of the whole bacterial cell lysates of these four strains yielded in characterization of the core proteome of *L. lactis* (586 proteins) that was responsible for stress resistance of this bacterium and its involvement in probiotic activities. The dynamic range of the core proteins was in four orders between the most and least abundant proteins. The gene ontology (GO) enrichment analysis revealed that the proteins identified in the core proteome were associated with translational processes. Strain-unique proteins were detected in strains NCDO2118 (38 proteins), IL1403 (15 proteins), and NZ9000 (11 proteins). In addition, the proteins not previously found in IL1403 (19 proteins) and MG1363 (3 proteins) strains were identified as well. The results of this work provided knowledge about the *L. lactis* functional proteome, which enhanced its future biotechnological potential [56].

Further, the probiotic properties of *Lactococcus lactis* subsp. *lactis* IL1403 were studied under ethanol-induced stress using the proteomic approach [57]. Over 400 extracted intracellular proteins were found to be significantly altered in response to ethanol, and among them, the proteins that were associated with general stress response, oxidative stress response, DNA repair, and ethanol metabolism were upregulated, in consistence with subsequent qRT-PCR analysis. In addition, various metabolic pathways were significantly enriched during ethanol stress to ensure survival of the cells and provide energy requirements to stressed *L. lactis* cells. The intracellular stress-related proteins enhanced the alcohol metabolism and activity of alcohol dehydrogenase (ADH), the process in which bioactive peptides were suggested to play essential roles in the past. The following study from the same group identified the molecular basis of the adaptive regulatory mechanism of *L. lactis* under ethanol-induced stress using both in silico prediction and in vivo validation in a mice model [58]. Herein, 18 ADH-activating peptides, derived and selected by various methods from the ethanol stress-related intracellular protein pool, were screened to select the proteins that were the sources of these ADH-activating peptides. The pentapeptide with sequence FAPEG exhibited the strongest ability to enhance ADH activity, and the interaction between FAPEG and ADH caused alteration in secondary structure of ADH. FAPEG exhibited the hepatoprotective effect and had the ability to prevent alcoholic liver injury (ALI) by, e.g., enhancing the activities of antioxidant and alcohol metabolism enzymes [58].

In another study, proteomic analysis of probiotics *Pediococcus pentosaceus* 1101 subjected to gastrointestinal conditions treatment in Man-Rogosa-Sharpe (MRS; BD Difco, Franklin Lakes, NJ, USA) medium with pH 3 and 0.5% bile salts for 1 h, revealed 100 proteins that were differentially expressed (73 and 27 proteins downregulated and upregulated, respectively), as compared to control (MRS with pH 7) [10]. Among these proteins that were upregulated or present only in a treatment group, signal transduction histidine-protein kinase ArlS (upregulated) and serine/threonine protein kinase (unique to a treatment group) were found. In addition, enzyme N-acetylmuramoyl-L-alanine amidase (32 kDa) was detected, which is known to be associated with antimicrobial activity. In summary, these proteins played crucial roles during the adaptation of *P. pentosaceus* 1101 in the gastrointestinal tract.

Recently, probiotic lactic acid bacteria subjected to different stress conditions during food processing and gastrointestinal transit (heat/cold, acid/alkaline, osmotic, oxidative, high hydrostatic pressure, and starvation stresses) were reviewed, and the importance of proteomics in the identification of protein changes was highlighted [59].

### 3.2. Surface Proteins Identification

Proteins on the surface of cells are among the first responders during the contact of probiotic bacteria with the environment/host. Surface proteins, including surface-anchored and surface-associated proteins, have essential roles in adhesion and subsequent colonization of probiotic organisms in the gastrointestinal tract [60]. The group of proteins on the bacterial surface (bacterial surface proteome/surfaceome) [19,61], totally or partially exposed on the external side of the cell membrane is crucial in communication between host cells and probiotic bacteria cells (cell-to-cell crosstalk), as well as among various bacterial probiotic strains, if multi-strain administration exhibited better benefit to the host [62]. The bacterial surfaceome is highly dynamic due to the constantly changing surrounding environment, and the analysis of surface proteins is challenging in many cases, as they are hydrophobic and can be of low abundances.

Probiotic bacteria produce a variety of surface proteins that are crucial in communication of bacteria with their host, and thus, they are often the targets for therapeutic purposes. Generally, in Gram-positive bacteria, surface proteins can be divided into five classes based on their attachment to the cell surface: (i) cell wall-anchored proteins (covalently bound at their C-terminal to the cell wall via motif LPXTG after reaction catalyzed by enzyme sortase; i.e., sortase-dependent proteins); (ii) non-covalently bound proteins (via interactions with binding domains of cell wall components, i.e., teichoic/lipoteichoic acids, glycopolymers); (iii) lipoproteins (anchored via their N-terminal); (iv) moonlighting proteins (multifunctional proteins that have multiple unique functions/activities [63,64]; e.g., glyceraldehyde 3-phosphate dehydrogenase (GAPDH) and enolase) and (v) macromolecular protein surface structures (S-layer, pilus, flagellum, and cellulosome) [5,61,65].

**Figure 3:**
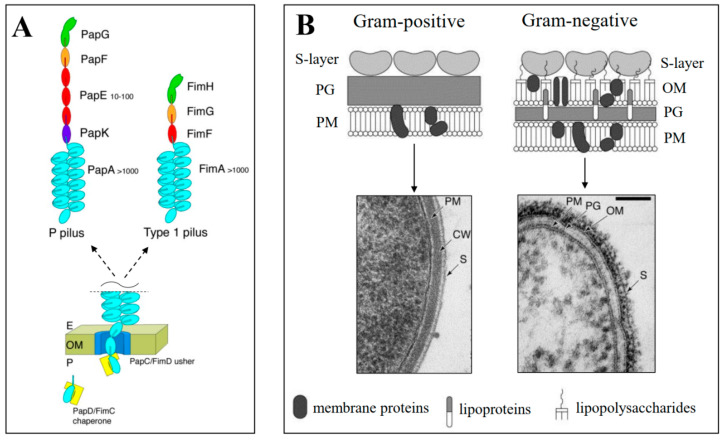
Pili and S-layers of bacterial cells. (**A**) Two types of multisubunit structures of *Escherichia coli* pili, P pilus, and type 1 pilus. P pilus and type 1 pilus tips contain PapG and FimH adhesin, respectively, that enable the attachment of bacteria to the receptors of epithelial cells of kidney (PapG) and bladder (FimH). PapG is connected through PapF adaptor subunit to fibrillum created by PapE homopolymer (10–100 copies) that is further attached to the pilus rod via PapK adapter subunit. In type 1 pilus, FimH is connected to one copy of FimG subunit and attached to the pilus rod via one copy of FimF subunit. The pilus rod includes a homopolymer of more than 1000 copies of PapA or FimA in P pilus and type 1 pilus, respectively. The bottom part of panel (**A**) shows schematically the chaperone/usher (CU) pathway that is responsible for pili assembly. CU pathway includes the noncovalent polymerization of pilus subunits bound to periplasmic chaperones (PapD and FimC for P pili and type 1 pili, respectively) at outer membrane (OM) assembly platform called usher. The usher catalyzes polymerization and enables the translocation of folded subunits across OM [33]; E—extracellular space; P—periplasmic space. (**B**) Schematic illustration and electron micrographs of cell envelope structures for Gram-positive and Gram-negative bacteria, both possessing cell-surface layer (S-layer). PG—peptidoglycan layer; PM—plasma membrane; CW—Gram-positive cell wall (usually, peptidoglycans compose the rigid wall); OM—outer membrane; S—S-layer. Figure 3A was adapted from ref. [33], and Figure 3B was adapted from ref. [34] with permission.

Many probiotic bacteria (e.g., genus *Lactobacillus*) have the outer part of the cell wall coated with a proteinaceous surface layer (S-layer), which is defined as an isoporous, symmetric, crystalline lattice-like layer containing self-assembly of monomeric proteins that are formed into a regularly spaced, two-dimensional array [66,67]. The proteins are called S-layer proteins (Slps), they are attached to carbohydrates of the cell wall by non-covalent interactions, and they can be associated with additional cell surface proteins SLAPs (S-layer associated proteins) [67,68]. In addition, many bacteria possess long, filamentous structures called pili that extend from their surfaces. Among the other purposes known, pili can serve as the means to overcome net repulsive forces caused by the negative charges of both bacterium cells and host cells during their attachment (adhesion) [69]. Pili are composed of several hundred 15–25 kDa protein subunits (pilins). In Gram-negative bacteria, pili are assembled by non-covalent homopolymerization of pilins, and in Gram-positive bacteria, pili are created by covalent polymerization of pilins under the presence of particular enzyme sortase [69]. For example, pili were recognized as effector molecules for *L. rhamnosus* GG [70] in relation to their immunomodulatory interactions with intestinal epithelial cells, and S-layer protein SlpA was found as an effector molecule for *L. acidophilus* NCFM during immune regulation [2,71]. Figure 3 (panel A) shows multisubunit structures of Gram-negative *E. coli* P pilus and type 1 pilus [33]. Figure 3 (panel B) shows a schematic illustration and electron micrographs of cell envelope structures for Gram-negative and Gram-positive bacterium with S-layers depicted [34].

The research on probiotic bacteria/host interactions can benefit from the data derived by proteomic experiments. Proteomics contributed to the knowledge about these interactions and underlying mechanisms at the protein level. It can help to uncover new therapeutic protein targets and treatment options. Early on, proteomics was shown to be a powerful method during the attempts at identification of cell wall proteomes of Gram-positive bacteria *Listeria* [72] and for prediction of the proteins that are potentially retained in the membrane of pathogenic bacteria *Bacillus subtilis* [73]. The additional proteomic studies have contributed to the clarification of the activities of probiotic bacteria surface proteins, e.g., in binding extracellular matrix proteins such as mucin, plasminogen, and fibronectin during adhesion to the gastrointestinal tract. For example, the proteomic approach has revealed five putative plasminogen-binding proteins in the cell wall of *Bifidobacterium lactis* [74]. Proteomics was used for analysis of outer surface-associated proteins from the probiotic organism *Lactobacillus plantarum* 299v [60]. Herein, the aim was to identify novel proteins included in adhesion and colonization of this bacterium in the gastrointestinal tract. This study revealed 29 proteins, including glycolytic proteins (e.g., GAPDH, phosphoglycerate kinase, enolase, and triose-phosphate isomerase), ribosomal proteins (e.g., elongation factor Tu and protein L12/L7), and stress-related proteins (e.g., heat shock proteins GrpE and DnaK). Many of these proteins were detected in the past on the outer surface of various *Lactobacillus* strains, and they showed the ability to bind intestinal mucosa components/cells as well as competitively exclude pathogenic bacteria [60]. A few examples of the newest studies that have been published in the past years are discussed below.

Using the MS-based proteomic approach, surface proteomes of *Lactobacillus crispatus* Lv25 and *Lactobacillus reuteri* RC14 were analyzed [75]. In these two strains, the presence of surface oligopeptide-binding proteins (OppA-like proteins) were confirmed, previously detected in *Lactobacillus salivarius* Lv72 during their participation as adhesins that recognized epithelial cell surface glycosaminoglycans. This indicated and confirmed the participation of OppA proteins in binding of diverse lactobacilli to the surface of human epithelial cells.

In another study, the authors aimed to characterize the cell adherence of the probiotic lactic acid bacterium *Pediococcus pentosaceus* GS4 strain to the epithelial cells in vitro [76]. Various methods, including MS, were used, and it was demonstrated that *P. pentosaceus* GS4 has the potential to produce 98 kDa surface layer protein Slp, which contributed to this cell adherence.

A quantitative MS-based shotgun proteomics was applied for identification of the patterns of S-layer proteins (Slps) and S-layer associated proteins (SLAPs) expressed by five probiotic *Levilactobacillus brevis* strains (PA6, A4, A7, M4, and DSMZ 20054) [67]. For the first time, 10 different Slps were identified on *Levilactobacillus brevis* (e.g., Slp M in addition to well-characterized Slps A, B, C, and D). Moreover, simultaneous detection of up to eight different Slps were found on the S layer of a single strain (A7). The numbers of reliably identified SLAPs were 106, 85, 129, 82, and 100 in A4, PA6, A7, M4, and in DSMZ 20054 strain, respectively, with 40 SLAPs common to the surface of all strains, albeit in various quantities. This work showed that these closely related strains exhibited the unique patterns of surface proteins, possibly causing their special functions.

Trypsin shaving and MS-based proteomics have been applied for detection of surface proteins in *Bifidobacterium longum* BBMN68 strain that were involved in bile stress response [77]. Out of 829 proteins with altered expression, 56 were upregulated (FC > 1.5) in response to bile stress, e.g., minor pilin subunit FimB was approximately 5-fold increased. Further, RT-PCR, scanning electron microscopy, and inhibition assays revealed that proteins FimA, FimB, and sortase srtC were involved in biosynthesis of sortase-dependent pili Pil1. Moreover, increased amount and length of Pil1 on *B. longum* BBMN68 was confirmed under bile stress. The experiments with inhibition of major pilin subunit FimA, which is known to be an adhesion component of Pil1, confirmed a crucial role of Pil1 in increased *B. longum* BBMN68 adhesion to HT-29 cells under this stress condition.

Food-grade *Lactobacillus casei* ATCC 393 was shown to synthesize Se nanoparticles (SeNPs) during the conversion of toxic sodium selenite into elemental selenium. This ability of several probiotic microorganisms was used as an important step in effective bioremediation, with removal of Se contamination and simultaneous production of SeNPs that were suitable for usage in biomedicine. Recently, the molecular mechanism of this ability was deciphered for *L. casei* ATCC 393 [78]. Herein, the treatment of cells by 4 mM sodium selenite yielded in the formation of spherical particles (65 ± 22 nm in size) with strong Se atom signal, as observed by energy dispersive spectrometer and transmission electron microscope, and the analysis of these particles, confirmed that *L. casei* ATCC 393 reduced selenite to elemental Se and compiled it into SeNPs. The particles were coated by surface proteins, and the analysis by LC-MS/MS identified various proteins (mainly in the range of 11–17 kDa) with the most abundant 50S ribosomal protein L7/L12 (12.6 kDa). It was demonstrated that surface proteins made SeNPs highly stable in solution. In addition, other key proteins associated with selenite reduction were determined, and it was concluded that *L. casei* ATCC 393 reduced selenite to SeNPs by glutathione- and nitrate reductase-mediated reduction pathway [78].

In 2019, a review article was published that discussed the important role of proteomics in the identification of probiotic surface-exposed proteins and the clarification of their involvement in probiotic action [19]. Probiotic and functional characteristics of *Ligilactobacillus (Lactobacillus) salivarius* strains were overviewed recently [79] in terms of their resistance to the conditions in the gastrointestinal tract, tolerance to acidic pH, adhesion to the intestinal mucosa, and their antioxidant and antibacterial properties against a variety of bacterial pathogens. In the same review, the roles of these beneficial bacteria examined by omics technologies were discussed, including genomics, transcriptomics, proteomics, and metabolomics [79]. 

### 3.3. Extracellular/Secreted Proteins Identification

The proteins secreted by bacterial cells and released into the extracellular space are termed extracellular/secreted proteins (exoproteomes/secretomes), and they mediate various cell interactions [80]. Basically, the proteins secreted into the extracellular medium can be identified/quantified by analyzing this medium by MS-based proteomic approaches. Using various cell culture conditions, the key proteins that regulate secretion machineries can be characterized in cell-conditioned media, and their molecular mechanisms can be determined. Additionally, the mechanism of cell-to-cell crosstalk can be investigated in co-cultures of various bacterial cells. However, many approaches for identification of secreted proteins are limited to in vitro experiments, which often do not reflect the situation in vivo. Thus, the development of new analytical and other techniques suitable for in vivo measurements of secreted protein origin, identity, and dynamics is of a high interest.

The experiments carried out in the past revealed that, e.g., conditioned medium derived from the probiotic *Lactobacillus rhamnosus* GG (naturally at pH 4) can modulate signal-transduction pathways of cultured murine intestinal epithelial cells [36]. Using various methods, including Western blotting and ELISA analyses, it was determined that the process was mediated by soluble low-molecular-weight peptides/proteins (acid and heat stable) that were secreted by *Lactobacillus rhamnosus* GG cells into conditioned medium, and they were shown to be active at pH 4 (inactive at pH 7). As well, ultrafiltration of conditioned media and treatment of epithelial cells by these filtrates showed that the bioactive peptides/proteins were in a range below 10 kDa. During treatment, the secreted peptides/proteins activated MAP kinases (ERK1/2, p38, and JNK) that further induced the expression of cytoprotective heat shock proteins Hsp25 and Hsp72 in intestinal epithelial cells. As further confirmed, the conditioned medium from *Lactobacillus rhamnosus* GG protected epithelial cells from oxidant damage, and it was suggested that one of the probiotics activities was the formation of cytoprotective heat shock proteins [36].

In another work, two novel proteins, p75 (75 kDa) and p40 (40 kDa), were purified from culture supernatant of *L. rhamnosus* GG and identified by MS-based proteomics [35]. By immunodepleting these proteins from *L. rhamnosus* GG-conditioned media, it was demonstrated that these proteins were able to activate protein kinase (Akt); they enhanced the cell growth and inhibited cytokine-caused epithelial cell damage and apoptosis in both human/mice colon epithelial cells and cultured mice colon explants. In this context, protein p40 showed more potent effect than p75. In summary, the proteins secreted by probiotic bacteria can prevent cytokine-modulated gastrointestinal disorders [35].

Probiotic bacteria *Limosilactobacillus reuteri* ZJ625 and *Ligilactobacillus salivarius* ZJ614 have shown improved benefits to the host when administered simultaneously in a multi-strain preparation, e.g., by modulation of the gut microbiome and having anti-inflammatory properties [62]. Therefore, to characterize the mechanism of intercellular crosstalk between these two species, the intracellular and extracellular proteomes were studied in single-cultures and co-culture and quantified by mass spectrometry. The analyses identified differentially expressed proteins in co-culture of these two strains compared to the cultures of each strain individually, with many post-translational modifications identified, such as oxidation, deamidation, ammonia loss, dehydration, and methylthiolation. In co-culture, a significant expression level of S-ribosylhomocysteine lyase was detected. S-ribosylhomocysteine lyase is known to be involved in synthesis of autoinducer-2, which is associated with previous interaction of probiotics with the microbiome. High levels of S-ribosylhomocysteine lyase in co-cultured strains indicated the crosstalk between *Limosilactobacillus reuteri* ZJ625 and *L. salivarius* ZJ614 mediated by S- ribosylhomocysteine lyase [62].

Further, exoproteome of probiotic *Lactobacillus mucosae* LM1 subjected to bile treatment (0.10% and 0.30% bile) was examined by label-free mass spectrometry analysis [81]. Under bile stress, the size of *L. mucosae* LM1 exoproteome increased in the bile response mechanism, and it contained secreted ribosomal proteins (e.g., 50S ribosomal proteins L27 and L16), metabolic proteins (e.g., lactate dehydrogenase, GAPDH, and pyruvate dehydrogenase), and various membrane-associated proteins as the key proteins. The differences in exoproteome composition (size and identities) as a result of *L. mucosae* LM1 treatment by various bile concentrations indicated that this probiotic bacterium regulated its exoproteome by secretion of intracellular proteins. The findings pointed out the proteins that *L. mucosae* LM1 cells used against bile stress in the gut [81].

The interaction between extracellular proteins secreted by probiotic bacteria and the human gut mucosa was reviewed in detail [82], and the review showed that extracellular proteins produced by probiotic strains of the genera *Bifidobacterium*, *Lactobacillus*, and *Escherichia* were mediators of effects that promoted mucosa-bacteria interactions, although these experiments were restricted mainly to in vitro arrangements. The different methods for investigation of protein secretion (e.g., fractionation-based assays and whole-cell-based assays) were overviewed, as well as the identification of secreted proteins by various bioinformatic tools and proteomics in vitro setting [46]. It was concluded that for analysis of bacterial secretion in a complex environment, e.g., within a living host, new tools are needed to accomplish this task [46].

### 3.4. The Proteins of EVs

The other mechanism to exert the beneficial functions of probiotic bacteria is via the cargo of their EVs [83]. Bacterial cells secrete EVs, the particles in the size of nanometers (<500 nm) enveloped by a lipid bilayer, that are released into the extracellular space as a response to various physiological and pathological conditions [84,85]. If Gram-positive bacteria releases EVs, EV lipid bilayer encloses cytosolic material, in the case of EVs secreted by Gram-negative bacteria, EV lipid bilayer encloses periplasmic material [9]. Similarly to EVs of eukaryotic cells, they can transfer encapsulated cargo (proteins, lipids, metabolites, and nucleic acids) to recipient cells, and the knowledge about their composition is essential for the characterization of associated biological functions and mechanisms [86,87]. EVs can be considered as postbiotics, i.e., secretory components of probiotic bacteria that confer health benefits [88,89], however, without the risk of infection caused by live microorganisms [90].

The recent study evaluated the protein composition of EVs derived from the probiotics *Lactobacillus plantarum*, *Lactobacillus fermentum*, and *Lactobacillus gasseri* by MS-based proteomics [91]. Herein, the EVs from *L. plantarum*, *L. fermentum,* and *L. gasseri* contained 98, 92, and 79 proteins, respectively. As the most abundant protein, GAPDH was identified in the EVs of *L. plantarum*, enolase in the EVs of *L. gasseri*, and the citrate lyase alpha chain in the EVs of *L. fermentum.* GAPDH is known to inhibit inflammation processes and can bind to human colonic mucin [91]. While the EVs derived from *L. fermentum* and *L. gasseri* were composed of many plasma membrane proteins, the EVs of *L. plantarum* contained more cytosolic proteins. As predicted by gene ontology (GO) enrichment analysis, the main molecular functions for the proteins of EVs were catalytic activity, binding, and transporter activity.

**Figure 4:**
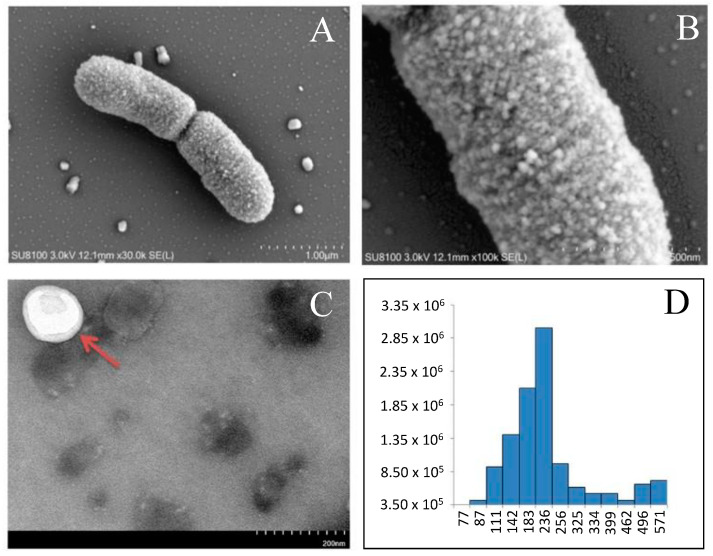
Transmission electron microscopy of *L. salivarius* SNK-6 strain with extracellular vesicles (EVs). (**A**) *L. salivarius* SNK-6 is a short, rod-shape bacterium arranged in pairs that releases nanosized EVs from the cell surface; (**B**) the magnification of the part of a bacterium; (**C**) the magnification of purified EV (pointing to by red arrow) that is spherical and enclosed by membrane; (**D**) the size distribution of the EVs that were released by *L. salivarius* SNK-6 and carried most of the cargo; EV diameters were from 111 to 256 nm, with the majority of EVs having diameters of 236 nm. This figure was adapted from ref. [92].

Other proteomic-based experiments were carried out on the EVs isolated from the culture media of *Lactobacillus salivarius* SNK-6 [92]. Herein, 320 proteins in total were identified (10–38 kDa) in the spherical EVs with diameters in the range of 100–256 nm, such as various anti-inflammatory proteins (elongation factor Tu, PrtP proteinase, chaperones). Determined by enrichment analysis, the EVs were composed of various functional proteins with key roles in signal transduction, energy metabolism, and biosynthesis. Figure 4 shows *L. salivarius* SNK-6 bacterium that releases spherical extracellular vesicles (EVs) from its surface, as visualized by transmission electron microscopy.

The core proteome of EVs purified from the concentrated supernatants of *Propionibacterium freudenreichii* CIRM-BIA129 were established using shotgun proteomics [93]. In past studies, it was shown that bacterial grow media (YEL—yeast extract lactate medium and UF—ultrafiltrate medium) can modulate the protein composition and biological functions of EVs that were purified by size-exclusion chromatography (SEC) [94]. In [93], ultracentrifugation (UC) was used for EVs purification from both YEL and UF media, and the EVs protein contents were compared to protein content of EVs purified by SEC as reported in [94]. The comparison of the EVs prepared by two purification methods (SEC and UC) and from two culture media (YEL and UF) showed that purification method was essential for the type and yield of the EVs. The EV core proteome (identified proteins common to all four conditions) consisted of 308 proteins. The differences in protein content were related rather to EVs purification methods (261 and 54 proteins were exclusive to EVs purified by UC and SEC, respectively) than to the type of culture media used. In conclusion, the selection of purification method was significant in obtaining different subpopulations of EVs with different protein contents [93].

The EVs from two probiotics, yeast *Saccharomyces boulardii* CNCM I-745 and bacterium *Streptococcus salivarius* K12, were isolated and their protein composition were identified [90]. By MS-based proteomics, 1641 and 466 proteins were detected in *S. boulardii* and *Streptococcus salivarius,* respectively. About 25% of all proteins identified in EVs of both species were metabolic proteins, with enzymes connected to cell wall rearrangement identified as well. The probiotic EVs were further tested in terms of their health-promotion applications and toxicity. It was observed that the EVs from both species stimulated the expression of interleukins IL-1β and IL-8 in human monocytic cells THP-1, and simultaneously, the EVs did not exhibit toxic features. In summary, the study demonstrated an efficient method for EVs isolation, and it contributed to the characterization of the protein composition of two diverse types of probiotics and their immunostimulatory effects [90].

Additionally, the biological effects of EVs released by various probiotic species were reviewed in [9]. Health-inducing properties and health-promoting effects of EVs secreted by non-pathogenic microorganisms and examined by different methods, including proteomics, were discussed [83]. Examples of the proteins of probiotic bacteria and in their EVs examined by proteomics in the period 2019–2024 are summarized in Table 1.

## 4. Health-Related Functions of Probiotic Bacteria Examined by Proteomics

The review articles on the health benefits of probiotics that targeted individual probiotic strains and species have been published for, e.g., *Lactobacillus rhamnosus* GG and *Bifidobacterium animalis* subspecies *lactis* BB-12 [6], *Lactobacillus casei* species [95], and *Ligilactobacillus salivarius* [79]. In addition, the systematic reviews and meta-analyses of randomized controlled trials to evaluate the effects of probiotics have been reported on depression [96], body weight in subjects with overweight or obesity [97], and prevention of allergy [98]. Probiotics-mediated suppression of cancer was discussed [99] and the application of proteomics to evaluate probiotics anticancer and chemopreventive abilities were described [100], as well as probiotic treatment for improvement of symptomatology in neurodegenerative disease [101]. Probiotics have been shown to modulate host intestinal microbiota during the prevention and treatment of arthritis [102]. The studies that reviewed the implementation of various omics technologies for the investigation of the mechanisms of pathology-induced responses of probiotic bacteria have been published, e.g., to unravel the mechanism of stress-response by integrating various omics platforms (genomics, transcriptomics, proteomics, metabolomics, and interactomics) [21].

Recently, the effects of probiotics (psychobiotics) *Bifidobacterium longum* Rosell^®^-175 (BB) and *Lactobacillus rhamnosus* JB-1 (LB) on brain proteome in mice were investigated [103]. Herein, psychobiotics were administered to mice groups (experimental BB and LB groups) for a total of 28 days, and differently expressed proteins in mouse hippocampus tissue were identified (13 proteins) as compared to the control group without psychobiotics. Among the identified proteins, transketolase (TK), syntaxin-binding protein 1 (STXB1), and eukaryotic translation initiation factor 4H (EIF4H) were downregulated in both experimental groups, and secreted frizzled-related protein 3 (SFRP3) and neuron-specific vesicular protein calcyon (CALCY) were upregulated in both BB and LB experimental groups. Glycine receptor subunit alpha-4 (GLRA4) was downregulated in BB group and upregulated in LB group. As well, odorant-binding protein 2 (OBP2) was downregulated in LB group compared to control, and carbonic anhydrase 2 (CAII) was downregulated in BB group as compared to LB group. In summary, the evidence of differentiation of proteomic profiles caused by the addition of psychobiotics into the mice diet was demonstrated in the mice brains. These psychobiotics increased the expression of proteins associated with maturation/activation of nerve cells and homeostatic modulation of neurogenesis; however, they decreased the levels of proteins related to central nervous system development and synaptic transmission. Thus, these results implied that psychobiotic bacteria can affect diverse neurological pathologies [103].

In another study, the reversion of intestinal inflammation in NCM460 cells by treatment with *Lactiplantibacillus plantarum* C9O4 was studied by proteomics [104]. Herein, control group, inflamed group (NCM460 cells exposed to inflammatory stimulus), and inflamed group pretreated with *Lpb. plantarum* C9O4 were compared. Proteomics identified over 1800 proteins. Out of them, 78.6% proteins were common to all three groups, and unique proteins were detected in control (4.7%), inflamed (2.8%), and inflamed pretreated (1.9%) groups. A total of 276 proteins significantly differed in the expression levels in NCM460 cells pretreated with *Lpb. plantarum* C9O4 as compared to the inflamed cell group; 173 and 103 proteins were downregulated and upregulated, respectively. In addition, the treatment of inflamed NCM460 cells by *Lpb. plantarum* C9O4 detected the decrease in expression levels of cytokines IL-2, IL-5, IL-6, and IFN-γ (interferon gamma) in inflamed cells. Further, the immunoregulatory role of *Lpb. plantarum* C9O4 in inflamed cells by stimulating the IFN-γ mediated JAK/STAT pathway was demonstrated. In conclusion, this study confirmed in vitro that *Lpb. plantarum* C9O4 is suitable for the regulation of chronic intestinal inflammatory disorders [104].

The response of probiotic bacterium *Bifidobacterium longum* subsp. *longum* GT15 to oxidative stress was evaluated after its exposure to hydrogen peroxide for 2 h and oxygen for 2 and 4 h using transcriptomic, proteomic, and metabolomic approaches [105]. By transcriptomics, more than 2-fold increases in transcript levels of 11 genes that encoded proteins with antioxidant activities were detected as compared to controls. Using proteomics, the expression of glutaredoxin and thioredoxin was upregulated when subjected to oxygen exposure, suggesting that the thioredoxin-dependent antioxidant pathway was involved in redox homeostasis of this bacterial strain. In addition, the levels of other proteins were found to be increased under these oxidative stress conditions, e.g., the levels of the proteins that were related to global stress and various types of metabolism. The data contributed to knowledge about the mechanism and factors that were related to the response of *B. longum* species to oxidative stress [105].

## 5. Concluding Remarks and Future Perspectives

The pros, cons, and many unknowns of probiotics have been discussed [106]. It was highlighted that clinical outcomes, whether beneficial or not, need to be evaluated carefully so that sensible decisions can be made. The better assessment of long-term safety of probiotics is needed in future clinical trials, especially in critically ill individuals, children, and immunosuppressed persons [107]. It was concluded recently that the safety of probiotic bacteria is strain-host-dose specific [108]. It was claimed that the safety of probiotics was not, in many instances, sufficiently tested and their consumption can exhibit adverse effects [109]. For example, probiotic bacteria *Lactococcus lactis* or *Lactobacillus casei* are generally considered safe microorganisms; however, they can exhibit diverse properties and pathogenicity. They were connected to mice mastitis [110], cases of bacteremia, sepsis, and to endocarditis of both healthy [111] and immunocompromised patients [112].

In addition, traditional probiotics from fermented products were replaced or applied simultaneously with dietary supplements in the form of pills and capsules, which enabled the consumption of increasing number of live microorganisms (10^10–12^) in one dosage. This, again, raises questions about their efficacy in particular illnesses if probiotic mixtures instead of personalized probiotic supplementation were applied, and especially, about the long-term safety of live bacteria intake. Therefore, recognition and isolation of bioactive proteins from probiotic cells and their future usage as effective therapeutics could allow the targeting of specific disorders in a controlled manner. Moreover, this way, the potential harm by live bacteria will be eliminated [36].

Although it was recognized that interactions between probiotics and the host is strain-dependent and the strain choice is of importance to exert specific effects to target a particular disorder, the precise mechanisms are still to be discovered in many cases. The individual native microbiota is an important factor for the treatment by probiotics, and interactions between the host microbiota and the specific bacterial strain need more examination for full utilization of their effects. The role of proteomics in the probiotics field at present and in the future can be seen in multiple ways: (i) the protein pattern identified via whole-cell protein profiling can be used for bacterial identification and strain typing; (ii) advanced proteomic techniques that determine fast dynamic changes in the proteins during treatment by different probiotic species/strains can contribute to the identification of a molecular basis of probiotic effects and species-specific functionality and mechanisms; (iii) proteomics can point out potential probiotic derived-proteins that are responsible for pro-health activities and the select probiotic strain that fulfills specific health requirements; this can lead to the production of agents for effective therapeutic interventions. In addition, proteomics that determined a unique proteome pattern linked to specific probiotic properties can be useful for the definition of molecular biomarkers for the selection of novel strains with predictable functionality [7]. MS-based proteomics can identify post-translational modifications (PTMs) that are crucial in the regulation of activities of proteins and control the dynamics of proteins under pathological events [113]. Thus, future probiotic proteomic studies should be targeted to the characterization of PTMs, especially PTMs of surface proteins, and their roles in crosstalk between probiotic cells and host cells.

As the study of the influence of probiotic bacteria on the host microbiome and on the modulation/regulation of various physiological/pathological conditions is getting increased attention, it is clear that the information provided by the single omics method is limited and insufficient. Thus, to obtain the desired answers on the characterization of complex probiotics activities and functions, the integrated multi-omics approach [114,115] is a promising direction in future experiments. It can include genomics, transcriptomics, proteomics, and metabolomics. For this reason, it is important to have the datasets from various omics experiments publicly available. Thus, the data can be integrated, novel information about molecular mechanisms can be accumulated between different molecular layers, and the molecules responsible for pathological outputs can be targeted. However, there are many challenges in omics data integration. For example, variable quality of the data used for integration is a major issue since it determines the accuracy and reliability of the results and enhances the data reproducibility. As the datasets are obtained from various sources and are measured at different experimental conditions, their integration can be difficult. Other issues can be datasets that have various formats and are composed of a large amount of data. In addition, unsynchronized annotations of transcriptomic and proteomic data can make the comparisons between coding regions and their expressed protein products difficult [116]. As well, when integrating a transcriptomics dataset and a proteomics dataset, the issue of mapping transcript identifiers to protein identifiers can be a problem since transcripts are translated into multiple protein isoforms. More details about the challenges and opportunities of integrative omics can be found in [116]. Several multi-omics data platforms, data integration pipelines, and tools for integrating multiple omics datasets have been introduced and recently reviewed [114]. As well, the application of omics methods that differ from traditional characterization methods expanded our knowledge about probiotics diversity and functionality. As they contribute to the whole picture in different ways, if used complementary, they create a powerful tool. Moreover, recent bioinformatics techniques with advanced methods of artificial intelligence are well suited for the processing of extensive data from the omics experiments. Recently, a combined proteomics, metabolomics, and in vivo analysis has been applied for the evaluation of both the functions and quality of probiotics in their large-scale production [117].

Further, confirmation is still needed, in many cases, whether the known probiotic effector molecules are responsible for the clinical outcomes observed in human trials. During the design of clinical trials, many aspects need to be taken into consideration, such as type of probiotic strain, its viability, dose, time frame for probiotic treatment, host site that needs to be targeted, etc. [2]. However, the quality of these trials differed, and low-quality evidence prevented the issuance of recommendations for particular probiotics to be applied in clinical practice. The results obtained from proteomic and other experiments helped to preselect the probiotic strain and point out potential protein targets for improvement of specific pathological conditions; however, they create the first steps in a whole process. The confirmation of health improvement in strong probiotics clinical trials with large patient cohorts is needed to bring the knowledge to the clinics in the near future. Recently, a technical review was published in which the American Gastroenterological Association Institute provided evidence-based information to guide both clinicians and patients regarding the use of probiotics for gastrointestinal diseases. It suggested which probiotic strains might be superior to others for particular disorders and which clinical contexts need additional research with probiotics [118].

Nowadays, efforts are directed toward the identification of new microbial strains of gut origin to develop next-generation probiotics (NGPs) for the prevention and treatment of human diseases/disorders [27,28]. This was possible with the progress in biotechnology (next-generation sequencing) and bioinformatics, that novel bacterial strains were identified in the gut microbiome that had no history of use as probiotics; they could fulfill their probiotic potential and could be used as live biotherapeutics or NGPs (“living microbes identified on the basis of comparative microbiome investigations which confer health advantages to their host when taken to suitable extents”) [119]. Whilst conventional probiotics are used in the form of functional foods and supplements, NGPs are considered as therapeutic drugs that possess pro-health features of specific bacterial strains and are regulated under the pharmaceutical framework [119]. NGPs as novel therapeutics have been discussed in detail [120], and using proteomics and artificial intelligence to introduce NGPs in personalized therapeutics has been overviewed [29].

## Figures and Tables

**Table 1:** Examples of the proteins in probiotic bacteria and in their EVs examined by proteomics in the period 2019–2024.

Probiotics	Type of Proteins Examined	Findings	Ref./ Year
*Pediococcus pentosaceus* 1101	intracellular proteins and whole-cell lysates	A total of 100 proteins were differentially expressed under exposure of the probiotic strain to 0.5% bile salts and acidity; two kinases and N-acetylmuramoyl-L-alanine amidase were identified as having crucial roles in adaptation of probiotic strain to conditions in the gastrointestinal tract.	[10]/ 2022
*Lactococcus lactis* subsp. *lactis* NCDO2118; *lactis* IL1403; *cremoris* NZ9000; *cremoris* MG1363	intracellular proteins and whole-cell lysates	The core proteome for *L. lactis* strains was determined (586 proteins) as responsible for strains stress resistance; strain-unique proteins were identified in three strains; 19 and 3 novel proteins to IL1403 and MG1363 strain, respectively, were found.	[56]/ 2019
*Lactococcus lactis* subsp. *lactis* IL1403	intracellular proteins and whole-cell lysates	Over 400 proteins were altered in the probiotic strain under ethanol-induced stress; stress-related proteins increased the activity of alcohol dehydrogenase.	[57]/ 2023
*Lactococcus lactis* subsp. *lactis* IL1403	intracellular proteins and whole-cell lysates	The molecular mechanism of ethanol-induced stress on the probiotic strain was determined using both in silico prediction and in vivo validation in a mice model. The FAPEG peptide exhibited the strongest ability to enhance alcohol dehydrogenase activity and prevent alcoholic liver injury.	[58]/ 2024
*Levilactobacillus brevis* strains PA6; A4; A7; M4; DSMZ 20054	surface proteins	Ten different S-layer proteins (Slps) were identified on these probiotic strains; simultaneous detection of eight Slps were found on the S-layer of A7 strain; 40 S-layer-associated proteins (SLAPs) were detected as common to all strains, however, with different quantities.	[67]/ 2022
*Lactobacillus crispatus* Lv25; *Lactobacillus reuteri* RC14	surface proteins	The presence of surface oligopeptide-binding proteins (OppA-like proteins) was confirmed in these strains; OppA proteins participated in the binding of diverse lactobacilli to the surface of human epithelial cells.	[75]/ 2019
*Pediococcus pentosaceus* GS4	surface proteins	The probiotic strain produced 98 kDa surface layer protein Slp that contributed to its adherence to epithelial cells in vitro.	[76]/ 2020
*Bifidobacterium longum* BBMN68	surface proteins	Out of 829 proteins identified with altered expression, 56 proteins were upregulated in the probiotic strain under a bile stress; the crucial role of pili (Pil1) was recognized in increased *B. longum* BBMN68 adhesion to HT-29 cells under bile stress conditions.	[77]/ 2024
*Lactobacillus casei* ATCC 393	surface proteins	Probiotic strain under sodium selenite treatment synthesized Se nanoparticles (SeNPs) by decreasing toxic sodium selenite into elemental selenium; SeNPs were coated by proteins in range 11–17 kDa with the most abundant 50S ribosomal protein L7/L12.	[78]/ 2023
*Limosilactobacillus reuteri* ZJ625; *Ligilactobacillus salivarius* ZJ614	extracellular/secreted proteins	Differentially expressed proteins were identified in co-culture of these two strains as compared to the cultures of each individual strain; the crosstalk between two strains in their co-culture was mediated by S-ribosylhomocysteine lyase.	[62]/ 2023
*Lactobacillus mucosae* LM1	extracellular/secreted proteins	The differences in exoproteome composition of probiotic strain under treatment by various bile concentrations indicated that this bacterium regulated its exoproteome by secretion of various intracellular proteins.	[81]/ 2021
*Lactobacillus plantarum*; *Lactobacillus fermentum*; *Lactobacillus gasseri*	proteins of EVs	As the most abundant protein, GAPDH was identified in the EVs of *L. plantarum*, the enolase in EVs of *L. gasseri*, and the citrate lyase alpha chain in the EVs of *L. fermentum*; EVs from *L. fermentum* and *L. gasseri* were composed of plasma membrane proteins, the EVs from *L. plantarum* contained more cytosolic proteins.	[91]/ 2023
*Lactobacillus salivarius* SNK-6	proteins of EVs	In total, 320 proteins were identified (10–38 kDa) in the spherical EVs with diameters in the range of 100–256 nm; various anti-inflammatory proteins were detected.	[92]/ 2024
*Propionibacterium freudenreichii* CIRM-BIA129	proteins of EVs	EVs from probiotics were prepared by two purification methods and from two different culture media; the differences in protein content were related rather to EVs purification methods than to the type of culture media used.	[93]/ 2023

## Data Availability

The data generated during the current study are available from the corresponding author on reasonable request.
